# Understanding HD Psychosis: An Analysis from the ENROLL-HD Database

**DOI:** 10.5334/tohm.395

**Published:** 2020-07-07

**Authors:** Ashwin Jaini, Jacob Yomtoob, Chen Yeh, Danny Bega

**Affiliations:** 1 Northwestern University Feinberg School of Medicine, Chicago, IL, US; 2 Department of Preventive Medicine, Division of Biostatistics, Feinberg School of Medicine, Northwestern University, Chicago, IL, US; 3 Department of Neurology, Northwestern University Feinberg School of Medicine, Chicago, IL, US

**Keywords:** Huntington Disease (HD), psychosis, enroll-hd

## Abstract

**Background:**

Psychosis is considered rare in Huntington’s Disease, with an estimated prevalence of 3–11%. However, it has a profound impact on quality of life and disease burden. This study uses the Enroll-HD database to determine the prevalence, onset, and severity of psychosis in Huntington’s Disease and to determine demographic and disease characteristics associated with psychosis.

**Methods:**

Data were obtained from Enroll-HD. Adults with manifest Huntington’s Disease were included. Descriptive statistics were calculated. Simple logistic regression was used to calculate the odds ratio with 95% confidence interval for association with each characteristic.

**Results:**

7,966 manifest Huntington’s Disease participants were analyzed, and 12.95% had a history of psychosis. Mean age of psychosis onset (48.34 years, SD 13.26) mirrored Huntington’s Disease onset. Family history of psychosis in a first degree relative was documented in 23.6% of participants with psychosis. Variables significantly (p < 0.05) associated with presence of psychosis in manifest HD included lower education level, unemployment, single marital status, depression, decreased verbal fluency score, and decreased total functional capacity & functional assessment score.

**Discussion:**

Psychosis in Huntington’s Disease is more prevalent than many prior studies have reported. It is associated with several demographic & psychiatric features, decreased cognitive capacity, and worse functional outcomes.

**Highlights:**

Psychosis in HD is more prevalent than prior studies have reported. It is associated with a range of demographic and psychiatric variables, worse cognition, and worse functional outcomes suggesting several features that may be used to predict onset of psychosis and improve understanding and management of psychosis in HD.

## Introduction

Huntington’s disease (HD) is an autosomal dominant neurodegenerative disease that has an incidence of about 0.38 per 100,000 individuals per year. The disease is characterized by progressive motor, psychiatric, and cognitive deterioration [1]. Although a large emphasis is placed on chorea and motor symptoms commonly seen at the time of diagnosis, the behavioral and mood changes associated with HD can be profoundly debilitating. Psychiatric abnormalities often begin early in HD, and can include irritability, affective disorders, apathy, and psychosis. Psychosis is considered relatively less common, with existing literature reporting a prevalence anywhere from 3–11% depending on the source [2,3,4,5,6,7]. Nevertheless, the impact of psychosis can be significant, dramatically reducing quality of life and increasing caregiver burden [8,9].

Data characterizing predictors of psychosis in HD are limited, likely due to its low prevalence compared to other psychiatric comorbidities. Most prior work on psychosis in HD is based on small/limited series which may underestimate the prevalence of psychosis, and treatment with antipsychotic medications taken for motor symptoms or irritability may mask milder psychotic symptoms from identification. Evaluation of psychosis in HD is further complicated by the limited ability of psychotic patients to participate in trials, necessitating use of large populations to better evaluate a significant number of people with HD and psychosis [10]. In 2018 Rocha et al. analyzed the frequency and factors associated with psychosis in the worldwide Enroll-HD cohort, which included 4,866 people with manifest HD [3]. However, since their analysis the Enroll-HD cohort has nearly doubled to include 7,988 people with manifest HD, allowing for a substantial analysis of psychosis to be undertaken.

The current study aims to better characterize the role of psychosis in HD using the largest HD dataset available to date with three primary goals in mind. Our first goal is to analyze prevalence of psychosis in HD. Existing data on prevalence of psychosis relies heavily on smaller studies, and even recent prevalence findings from Rocha et. al. represent a sample size much smaller than currently available. Our second goal is to measure psychosis severity as well as age of psychosis onset relative to age of HD diagnosis, in order to better characterize the nature and onset of psychosis within this population. Our third goal is to better understand demographic and disease characteristics that are associated with psychosis as a means of being better able to predict risk, screen, and manage HD psychosis. This portion of the analysis will reexamine previous findings of Rocha et. al. in a significantly larger sample size, and will provide novel information on other characteristics that are clinically relevant but have not been previously studied.

## Methods

### Participants

The current study includes data obtained from Enroll-HD, a longitudinal prospective observational research platform designed to better understand HD onset and progression. At the time of this analysis (December 2018) Enroll-HD included over 15,000 participants from 29 countries worldwide [11]. Participants from the Enroll-HD database who were classified as having manifest HD (exhibiting signs or symptoms of disease) at enrollment were included in the current study. Participants who were classified as having pre-manifest HD (gene expansion carriers without signs or symptoms of disease) or as genotype negative were excluded from the current study. Participants with an HD onset age below 18 or a CAG repeat length over 70 were excluded as outliers.

### Variables

All data regarding variables of interest were obtained from each participant’s baseline visit. Positive history of psychosis was used for each included participant to create a psychosis subgroup, based on the rater’s response to the question “Has psychosis (hallucinations or delusions) ever been a part of the participant’s medical history?” at baseline visit. For severity of psychosis, Problem Behaviours Assessment Form (Short PBA) Psychosis Group Domain score was used for each participant at baseline visit. These variables addressed our first and second major goals.

With our third aforementioned goal in mind, we chose variables a priori as potentially meaningful predictors of psychosis from the available data in the Enroll-HD dataset. Demographic, psychiatric, cognitive, and functional variables that were thought to be the most clinically relevant to HD psychosis presentation, quality of life, and management strategy were included for analysis. Demographic variables used from baseline visit included gender, ethnicity, family history of HD, marital status, International Standard Classification of Education (ISCED), employment, initial major symptom noted by participant, rater’s judgment of initial major symptom, age of motor symptom onset, age of clinical HD diagnosis, and larger research CAG allele. Psychiatric variables used from baseline visit included previous suicidal ideation/attempts, history of depression, history of irritability, history of violent/aggressive behavior, history of apathy, history of perseverative obsessive behaviors, history of cognitive impairment or dementia, history of alcohol abuse, number of pack-years smoked, history of drug abuse, depression PBA score, irritability/aggression PBA score, apathy PBA score, executive function PBA score, anxiety subscore (Hospital Anxiety and Depression Scale – Snaith’s Irritability Scale (HADS-SIS)), depression subscore (HADS-SIS), irritability subscore (HADS-SIS), outward irritability subscore (HADS-SIS), and inward irritability subscore (HADS-SIS). Functional and cognitive variables used from baseline visit included BMI, total motor score (TMS), timed up and go (TUG), total functional capacity (TFC), functional assessment score (FAS), symbol digit modality test (SDMT)- total correct, verbal fluency- total correct 1 min, Stroop colour naming test- total correct, and Mini Mental Status Exam (MMSE) score.

### Data Analysis

Categorical variables were summarized with use of counts and percentages, and continuous variables with means and standard deviations or median and interquartile range as appropriate. The primary variable of scientific interest was the presence of psychosis. Simple logistic regression with presence of psychosis as binary outcome was used to calculate the odds ratio with 95% confidence interval for association with predictor one-at-a-time. To perform this, we put each predictor into the model one at a time and then used the model to compute the odds ratio and its corresponding 95% confidence interval. Benjamini-Hochberg procedure was used to decrease type I errors and adjust for multiple comparisons. All statistical analysis was performed in SAS version 9.4 or R 3.3.3.

## Results

**Figure 1:**
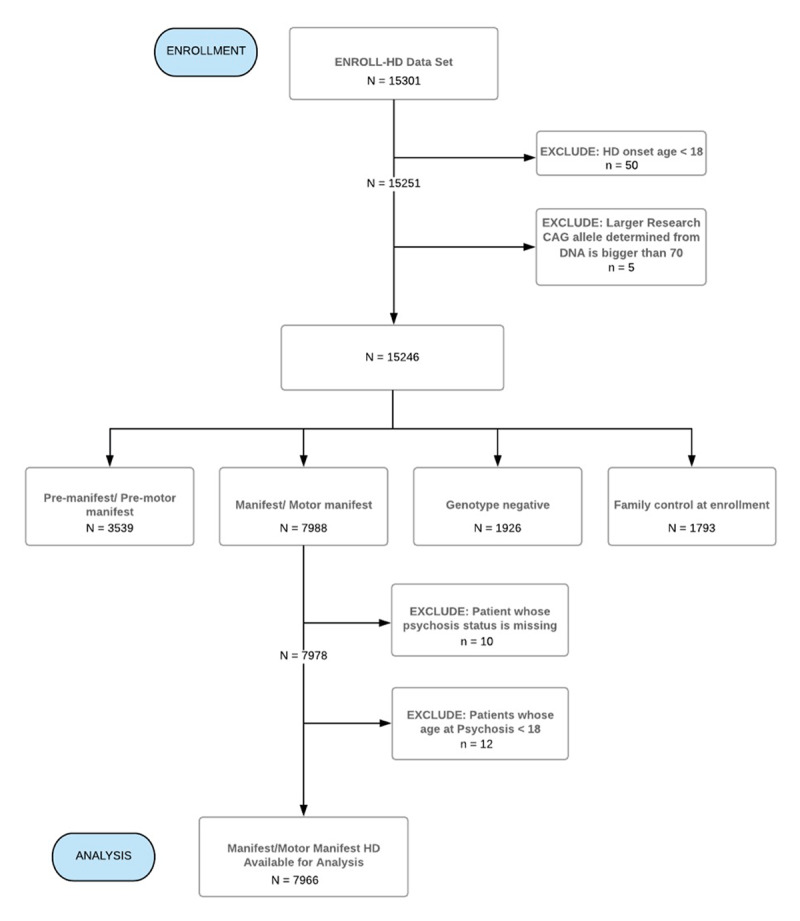
**Consort Diagram for HD Psychosis Analysis Group Selection.** Inclusion & exclusion criteria and breakdown of participant group included in analyses of psychosis in HD. In addition to non-manifest/motor-manifest HD participants being excluded, those with an HD onset age < 18 or a CAG repeat length > 70 were also omitted. Among the 7988 manifest/motor-manifest HD participants remaining, 10 participants were excluded due to missing data on psychosis status, and 12 participants were excluded due to childhood onset of psychosis. Our final analysis group included 7966 manifest/motor-manifest HD participants, of which 1021 had psychosis and 6945 did not.

Figure 1 demonstrates selection of Enroll-HD participants and inclusion in this study. 7,966 manifest HD participants were included and 1,033 (12.95%) had a history of psychosis. In the pre-manifest group of 3,539 participants, 100 (2.83%) had a history of psychosis. Mean age of onset of psychosis in the manifest HD psychosis subgroup was not significantly different than mean age of HD diagnosis in the entire manifest HD cohort (48.34 years (SD 13.26) vs. 48.81 years, p = .293). Analysis of psychosis severity by PBA psychosis score showed an average score of 2.215, reflecting mild to moderate psychosis severity on average. 23.6% of manifest HD participants with psychosis had a positive family history of psychosis in a first degree relative.

Bivariate analysis was performed to evaluate the association between different demographic & disease characteristics and presence of psychosis in manifest HD. Several factors were significantly (p < 0.05) associated with presence of psychosis in the manifest HD study group (Tables 1 and 2). In Tables 1 and 2, all significant p-values remained significant after Benjamini-Hochberg procedure and are denoted as * in the table.

**Table 1:** Bivariate Analysis of Baseline Characteristics Associated with HD Psychosis.

Characteristic	Psychosis – NO	Psychosis – YES	Logistic Regression	

	Mean (SD)/Median (Q1,Q3) or N (%)	Mean (SD)/Median (Q1,Q3) or N (%)	OR (95% CI)	P-value

Male Sex	3376 (48.61)	491 (48.09)	0.98 (0.86,1.12)	0.76
‘Caucasian’ Ethnicity	6542 (94.24)	953 (93.43)	0.87 (0.67,1.15)	0.31
Previous Suicidal Ideation/Attempt	1887 (27.2)	463 (45.62)	2.24 (1.96,2.57)	<0.01*
Positive Family History of HD	5792 (83.4)	907 (88.83)	1.58 (1.30,1.95)	<0.01*
Marital Status				
Married/Partnership	4371 (63.02)	584 (57.25)	reference	
Other	1313 (18.93)	207 (20.29)	1.18 (0.99,1.40)	0.06
Single	1252 (18.05)	229 (22.45)	1.37 (1.16,1.61)	<0.01*
ISCED Education Level 1 of 4,5,6	3037 (43.94)	323 (31.92)	0.60 (0.52,0.69)	<0.01*
Unemployed	5389 (77.81)	929 (91.17)	2.94 (2.37,3.71)	<0.01*
Initial Major Symptom Noted By Participant				
Motor	4051 (61.91)	429 (46.48)	reference	
Cognitive	739 (11.29)	77 (8.34)	0.98 (0.76,1.26)	0.90
Psychiatric	1038 (15.86)	295 (31.96)	2.68 (2.28,3.16)	<0.01*
Other/Mixed	715 (10.93)	122 (13.22)	1.61 (1.29,1.99)	<0.01*
Rater’s Judgment of Initial Major Symptom				
Motor	3761 (54.71)	342 (34.58)	reference	
Cognitive	586 (8.52)	60 (6.07)	1.13 (0.84,1.49)	0.42
Psychiatric	1260 (18.33)	365 (36.91)	3.19 (2.71,3.74)	<0.01*
Other/Mixed	1267 (18.43)	222 (22.45)	1.93 (1.61,2.31)	<0.01*
Positive History of Depression	4877 (70.24)	868 (85.27)	2.45 (2.05,2.95)	<0.01*
Positive History of Irritability	4548 (65.50)	864 (84.87)	2.95 (2.48,3.54)	<0.01*
Positive History of Violent/Aggressive Behavior	2372 (34.16)	677 (66.57)	3.84 (3.34,4.42)	<0.01*
Positive History of Apathy	4202 (60.51)	842 (82.71)	3.12 (2.64,3.71)	<0.01*
Positive History of Perseverative/Obsessive Behavior	3454 (49.75)	764 (75.35)	3.09 (2.66,3.59)	<0.01*
History of Significant Cognitive Impairment/Dementia	3948 (56.90)	811 (79.51)	2.94 (2.51,3.45)	<0.01*
History of Alcohol Abuse	635 (9.17)	163 (16.06)	1.90 (1.57,2.28)	<0.01*
History of Drug Abuse	615 (8.88)	121 (11.93)	1.39 (1.13,1.70)	<0.01*
Number of Pack-years smoked in Participant’s History	16.8 [7.0,30.0]	18.0 [8.0,31.5]	1.01 (1.00,1.01)	0.02*
Age of Motor Symptom Onset	46.33 (11.96)	44.84 (11.92)	0.99 (0.98,1.00)	<0.01*
Age of Clinical HD Diagnosis	48.97 (12.38)	47.69 (12.41)	0.99 (0.99,1.00)	<0.01*

Bivariate analysis of baseline characteristics showed a range of demographic (positive family history of HD, single marital status, ISCED education level, unemployed), psychiatric history (previous suicidal ideation/attempt, drug/alcohol abuse, depression, irritability, aggression, perseverative/obsessive behavior, apathy, anxiety) and clinical history (age of motor symptom onset, age of clinical HD diagnosis, and initial major symptom noted by participant and rater) variables all associated with the presence of psychosis in manifest/motor-manifest HD. * for p-value means remaining significant after Benjamini-Hochberg procedure.

**Table 2:** Bivariate Analysis of Clinical Assessment Scores Associated with HD Psychosis.

Characteristic	Psychosis – NO	Psychosis – YES	Logistic Regression		

	Mean (SD)/Median (Q1,Q3) or N (%)	Mean (SD)/Median (Q1,Q3) or N (%)	OR (95% CI)	P-value

BMI	24.92 (4.97)	25.15 (5.51)	1.01 (1.00,1.02)	0.18
Total Motor Score	37.36 (21.14)	48.15 (25.13)	1.02 (1.02,1.02)	<0.01*
TUG Test^1^- Total Time	10.00 [8.00,12.00]	11.00 [8.22,15.00]	1.03 (1.02,1.04)	<0.01*
TFC Score^2^	9 [6,11]	5 [2,8]	0.83 (0.81,0.84)	<0.01*
Functional Assessment Score	20 [15,24]	14 [7,19]	0.91 (0.90,0.92)	<0.01*
SDMT Test^3^- Total Correct	23.49 (13.01)	16.20 (12.53)	0.95 (0.95,0.96)	<0.01*
Verbal Fluency- Total Correct 1 min	12.23 (5.84)	9.40 (6.01)	0.91 (0.90,0.93)	<0.01*
Stroop Color Naming Test- Total Correct	42.34 (17.82)	32.36 (18.91)	0.97 (0.97,0.97)	<0.01*
MMSE^4^ Score	26 [23,28]	24 [19,27]	0.90 (0.89,0.92)	<0.01*
Depression PBA^5^ Score	3 [0,8]	5 [1,12]	1.04 (1.03,1.05)	<0.01*
Irritability Aggression PBA Score	1 [0,4]	2 [0,8]	1.06 (1.05,1.07)	<0.01*
Apathy PBA Score	1 [0,6]	4 [0,9]	1.10 (1.09,1.12)	<0.01*
Executive Function PBA Score	0 [0,5]	4 [0,11]	1.09 (1.08,1.10)	<0.01*
Anxiety HADS-SIS^6^ Subscore	5 [3,9]	6 [3,10]	1.06 (1.03,1.08)	<0.01*
Depression HADS-SIS Subscore	6 [3,9]	7 [3,10]	1.06 (1.04,1.09)	<0.01*
Irritability HADS-SIS Subscore	5.00 [2.00,9.00]	6.00 [2.75,10.00]	1.03 (1.01,1.05)	0.01*
Outward Irritability HADS-SIS Subscore	3 [1,6]	4 [1,6]	1.03 (1.00,1.06)	0.09
Inward Irritability HADS-SIS Subscore	2 [0,4]	2 [0,4]	1.05 (1.01,1.09)	0.01*
Larger Research CAG Allele Determined from DNA	43.94 (3.73)	44.16 (3.92)	1.02 (1.00,1.03)	0.08

Bivariate analysis of clinical assessment scores showed a range of cognitive (SDMT, Verbal Fluency, Stroop Color Naming Test, MMSE Score), psychiatric (Depression PBA score, Irritability/aggression PBA score, Apathy PBA score, Executive function PBA score, Anxiety HADS-SIS Subscore, Depression HADS-SIS Subscore, Irritability HADS-SIS Subscore, Inward Irritablity HADS-SIS Subscore) and functional variables (Total Motor Score, TUG Test, TFC Score, Functional Assessment Score) all associated with presence of psychosis in manifest/motor-manifest HD. * Abbreviations: ^1^ Timed Up and Go Test; ^2^ Total Functional Capacity; ^3^ Symbol Digit Modality Test; ^4^ Mini Mental Status Exam; ^5^ Problem Behavior Assessment; ^6^ Hospital Anxiety and Depression Scale & Snaith Irritability Scale. * for p-value means remaining significant after Benjamini-Hochberg procedure.

## Discussion

This study represents the largest analysis of HD psychosis that has been conducted to date, and presents new information on prevalence, severity, onset age, family history, and associated factors of psychosis in a large, manifest HD population. In particular, our findings regarding psychosis prevalence as well as demographic & disease correlates offer new insight into HD psychosis with potential clinical implications.

With regards to prevalence, 12.95% of participants with manifest HD had a history of psychosis, while only 2.83% of participants with pre-manifest HD had a history of psychosis. Pre-manifest findings parallel the 3% psychosis rate previously reported in the general population [12]. However, our manifest HD results indicate a higher prevalence than previously reported in manifest HD studies. Notably, Rocha et. al. had reported a psychosis prevalence of 10.8% in their 2018 analysis using an earlier iteration of the Enroll-HD database, more than 2% lower than these results [3]. We believe this increase is likely due to the large increase in sample size in the current study (7,966 vs 2,303 manifest HD participants used to calculate prevalence). Taken in the context of this as well as other recent studies, the current study shows that psychosis is more prevalent in manifest HD than previously predicted.

Regarding our bivariate analysis, several demographic, psychiatric, cognitive, and functional variables were positively associated with psychosis in manifest HD. In their 2018 analysis of manifest participants in the Enroll-HD cohort (n = 4,866), Rocha et al. found several factors associated with psychosis including younger age at clinical HD diagnosis, historical features (alcohol use disorder, depression, violent/aggressive behavior, perseverative/obsessive behavior), and cognitive/functional assessments (lower TFC score, longer time to complete trail making test-B) [3]. In addition to confirming and strengthening several of these associations with a larger sample size (n = 7,966), the current study offers new information on several previously unexplored variables associated with psychosis in HD.

Regarding demographic variables, presence of psychosis was associated with a mix of features, including single marital status (OR 1.37), lower ISCED education levels (OR 0.60), unemployment (OR 2.94), younger age of clinical HD diagnosis (OR 0.99), and younger age of motor symptom onset (OR 0.99) (Table 1). Aside from younger age of clinical HD diagnosis [3], these demographic findings have not been previously described. With regards to single marital status, research has tied lack of consistent social engagement to increased likelihood of psychiatric conditions such as psychosis, so it is possible these findings reflect the impact of decreased social stimulation [13]. With regards to education level, an association between decreased education level and increased psychotic symptoms has already been described, so the current study suggests that this association persists within HD as well [14]. Of note, these findings are associative rather than causative, as it is seemingly just as likely that psychosis and other psychiatric manifestations may be responsible for these demographic findings in this population. Regarding age of clinical HD diagnosis and age of motor symptom onset, the current study strengthens the assertion that earlier onset age of HD even at similar manifest stage may portend higher likelihood of psychosis, particularly in those who exhibit early motor symptoms.

Regarding psychiatric features, psychosis in manifest HD was associated with positive history of all analyzed measures, including previous suicidal ideation/attempt (OR 2.24), depression (OR 2.45), irritability (OR 2.95), violent/aggressive behavior (OR 3.84), apathy (OR 3.12), perseverative/obsessive behavior (OR 3.09), and alcohol/drug abuse (OR 1.90/1.39) (Table 1). These findings strengthen previous findings regarding psychiatric conditions and psychosis by reproducing them in a larger manifest HD group [3,10], and were largely expected due to the close relationship between psychosis and these conditions.

Psychosis in HD also correlated with a general decrease in cognitive capacity, shown by a significant decrease in SDMT (OR 0.95), verbal fluency test total correct (OR 0.91), Stroop Color Naming Test total correct (OR 0.97), and MMSE scores (OR 0.90) (Table 2). The correlation between psychosis and cognitive impairment has been well characterized, and our findings add to existing research supporting this association within manifest HD [3,15].

Finally, psychosis was associated with decreased functional ability, marked by increases in Total Motor Score (OR 1.02) and TUG Test total time (OR 1.03) and decreases in TFC Score (OR 0.83) and Functional Assessment Score (OR 0.91) (Table 2). While psychosis has previously been linked to changes in TMS and TFC scores [3,10], the current study utilizes new functional variables (TUG Test and FAS) in addition to reproducing previous findings in a larger manifest HD group. Our findings suggest a more robust association between psychosis and decreased functional capacity in manifest HD.

With regards to age of onset, there was no significant difference between mean age of onset of psychosis in our psychosis subgroup (48.34 years) and age of recognized HD onset (48.81 years) within the larger manifest group. This supports the current understanding that psychiatric symptoms may often arise early in HD progression, as opposed to exclusively being a late-stage outcome [16]. Rocha et al. reported that psychotic symptoms preceded clinical diagnosis in >50% of participants [3]. However, this interpretation is limited by the fact that those with more advanced disease may be unable to complete Enroll-HD assessments or may not attend clinic, thereby skewing the population toward younger, healthier people with HD.

Mean severity of psychosis based on Psychosis Group Domain PBA score indicated mild to moderate severity of psychosis (mean = 2.215) in manifest HD, supporting findings from previous studies [10]. Additionally, family history of psychosis in a first degree relative was positive in 27.5% of our psychosis HD subgroup, which represents a higher ratio than that reported in non-HD populations [17]. Our findings suggest that family history may be a better predictor for psychosis in HD as opposed to the general population, and supports prior research on familial aggregation of psychosis in HD [18].

Taken in total, the results from the current study offer new insight that could inform clinical practice regarding identification and management of psychosis in manifest HD. Increased psychosis prevalence and early age of onset of psychosis in HD may encourage earlier clinical investigation of psychosis in manifest HD individuals, especially those with motor symptoms at an earlier age. Similarly, manifest HD patients with other psychiatric presentations or family history of psychosis may benefit from an earlier evaluation for possible psychosis given the clear associations described in this study. Demographic factors could indicate possible areas of emphasis for lifestyle changes to affect psychosis in manifest HD – for example, the association between single marital status and psychosis in manifest HD suggests the importance of social engagement, and emphasizing this in management strategy could prove beneficial for manifest HD patients with psychosis. Finally, the current study emphasizes the need to address functional status in management plans for manifest HD individuals with psychosis. Assistance with activities of daily living & functional adaptations could provide significant benefit to quality of life in this patient population.

### Limitations and Future Directions

The primary limitation of our analysis is that it is correlational and causative relationships cannot be determined. The primary strength of our study is the large sample size. However, as a retrospective study, the data included in our analysis is subject to recall bias, recruitment bias, and our study offers no information on treatment. We reported the percentage of patients with history of psychosis rather than those with active psychosis as identified by the PBA-psychosis score. This allowed identification of a larger affected patient population at the expense of analysis being limited by bias inherent in patient self-reporting. Participants with more severe psychiatric manifestations may have been less inclined to participate and may actually lead to an under-estimate of our findings. Another limitation of our study is that subjects with and without psychosis were not matched based on disease severity beyond manifest HD status. Since the psychosis group had an earlier average age of disease onset, this likely impacted the overall findings of more severe disease manifestations in most variables. This study strengthens our understanding of psychosis as manifestation of more advanced disease. Our study therefore better describes the clinical characteristics of the HD population with psychosis but cannot fully determine which variables correlate with psychosis in isolation. Multivariate analysis was not conducted because we did not aim to develop a predictive model, though future studies may use variables identified here in developing multivariate predictive models. Future longitudinal, prospective, and matched studies would allow for better characterization of the presentation, evolution, and treatment of psychosis in people with HD and for isolation of characteristics that correlate with psychosis.

## Conclusion

This study expands upon the findings of prior smaller analyses of factors associated with psychosis in HD. Psychosis in manifest HD is more prevalent than prior studies have reported. It is associated with a range of demographic and psychiatric variables, worse cognition, and worse functional outcomes suggesting several features that may be used to predict onset of psychosis and improve understanding and management of psychosis in HD.

## References

[1] Labbadia J, Morimoto RI. Huntington’s disease: underlying molecular mechanisms and emerging concepts. Trends in biochemical sciences. 2013; 38(8): 378–85. DOI: 10.1016/j.tibs.2013.05.003 23768628 PMC3955166

[2] van Duijn E, Kingma EM, van der Mast RC. Psychopathology in verified Huntington’s disease gene carriers. The Journal of neuropsychiatry and clinical neurosciences. 2007; 19(4): 441–8. DOI: 10.1176/jnp.2007.19.4.441 18070848

[3] Rocha NP, Mwangi B, Gutierrez Candano CA, Sampaio C, Furr Stimming E, Teixeira AL. The Clinical Picture of Psychosis in Manifest Huntington’s Disease: A Comprehensive Analysis of the Enroll-HD Database. Frontiers in neurology. 2018; 9: 930 DOI: 10.3389/fneur.2018.00930 30459704 PMC6232301

[4] Paoli RA, Botturi A, Ciammola A, et al. Neuropsychiatric Burden in Huntington’s Disease. Brain Sci. 2017; 7(6): 67 Published 2017 6 16 DOI: 10.3390/brainsci7060067 PMC5483640 28621715

[5] Craufurd D, Thompson JC, Snowden JS. Behavioral changes in Huntington Disease. Neuropsychiatry Neuropsychol Behav Neurol. 2001 Oct-Dec; 14(4): 219–26. 11725215

[6] Murgod UA, Saleem Q, Anand A, Brahmachari SK, Jain S, Muthane UB. A clinical study of patients with genetically confirmed Huntington’s disease from India. J Neurol Sci. 2001 9 15; 190(1–2): 73–8. DOI: 10.1016/S0022-510X(01)00593-7 11574110

[7] Paulsen JS, Ready RE, Hamilton JM, Mega MS, Cummings JL. Neuropsychiatric aspects of Huntington’s disease. J Neurol Neurosurg Psychiatry. 2001 9; 71(3): 310–4. DOI: 10.1136/jnnp.71.3.310 11511702 PMC1737562

[8] Flyckt L, Lothman A, Jorgensen L, Rylander A, Koernig T. Burden of informal care giving to patients with psychoses: a descriptive and methodological study. The International journal of social psychiatry. 2013; 59(2): 137–46. DOI: 10.1177/0020764011427239 22100570 PMC3652598

[9] Melle I, Friis S, Haahr U, Johannesen JO, Larsen TK, Opjordsmoen S, et al. Measuring quality of life in first-episode psychosis. European psychiatry: the journal of the Association of European Psychiatrists. 2005; 20(7): 474–83. DOI: 10.1016/j.eurpsy.2005.03.002 15967642

[10] van Duijn E, Craufurd D, Hubers AA, Giltay EJ, Bonelli R, Rickards H, et al. Neuropsychiatric symptoms in a European Huntington’s disease cohort (REGISTRY). Journal of neurology, neurosurgery, and psychiatry. 2014; 85(12): 1411–8. DOI: 10.1136/jnnp-2013-307343 24828898

[11] Landwehrmeyer GB, Fitzer-Attas CJ, Giuliano JD, Goncalves N, Anderson KE, Cardoso F, et al. Data Analytics from Enroll-HD, a Global Clinical Research Platform for Huntington’s Disease. Mov Disord Clin Pract. 2017; 4(2): 212–24. DOI: 10.1002/mdc3.12388 30363395 PMC6174428

[12] Perala J, Suvisaari J, Saarni SI, Kuoppasalmi K, Isometsa E, Pirkola S, et al. Lifetime prevalence of psychotic and bipolar I disorders in a general population. Archives of general psychiatry. 2007; 64(1): 19–28. DOI: 10.1001/archpsyc.64.1.19 17199051

[13] Wang J, Lloyd-Evans B, Giacco D, Forsyth R, Nebo C, Mann F, et al. Social isolation in mental health: a conceptual and methodological review. Social psychiatry and psychiatric epidemiology. 2017; 52(12): 1451–61. DOI: 10.1007/s00127-017-1446-1 29080941 PMC5702385

[14] Barch D, Sheffield J. Cognitive impairments in psychotic disorders: common mechanisms and measurement. World Psychiatry. 2014 10; 13(3): 224–232. DOI: 10.1002/wps.20145 25273286 PMC4219054

[15] Swanson CL, Jr., Gur RC, Bilker W, Petty RG, Gur RE. Premorbid educational attainment in schizophrenia: association with symptoms, functioning, and neurobehavioral measures. Biological psychiatry. 1998; 44(8): 739–47. DOI: 10.1016/S0006-3223(98)00046-8 9798078

[16] Julien CL, Thompson JC, Wild S, Yardumian P, Snowden JS, Turner G, et al. Psychiatric disorders in preclinical Huntington’s disease. Journal of neurology, neurosurgery, and psychiatry. 2007; 78(9): 939–43. DOI: 10.1136/jnnp.2006.103309 PMC2117854 17178819

[17] Faridi K, Pawliuk N, King S, Joober R, Malla AK. Prevalence of psychotic and non-psychotic disorders in relatives of patients with a first episode psychosis. Schizophrenia research. 2009; 114(1–3): 57–63. DOI: 10.1016/j.schres.2009.07.007 19666214

[18] Tsuang D, Almqvist EW, Lipe H, Strgar F, DiGiacomo L, Hoff D, et al. Familial aggregation of psychotic symptoms in Huntington’s disease. The American journal of psychiatry. 2000; 157(12): 1955–9. DOI: 10.1176/appi.ajp.157.12.1955 11097960

